# First Molecular Detection and Genetic Characterization of *Tetratrichomonas buttreyi* and *Pentatrichomonas hominis* in Donkeys in Shanxi Province, China

**DOI:** 10.3390/ani14182651

**Published:** 2024-09-12

**Authors:** Han-Dan Xiao, Shuo Zhang, Yi-Han Lv, Ze-Dong Zhang, Nan Su, Liang-Liang Li, Xing-Quan Zhu, Shi-Chen Xie, Wen-Wei Gao

**Affiliations:** 1 Laboratory of Parasitic Diseases, College of Veterinary Medicine, Shanxi Agricultural University, Jinzhong 030801, China; 15166873600@163.com (H.-D.X.); shuoshuozhang0828@163.com (S.Z.); yh001028@126.com (Y.-H.L.); zzd18203541696@163.com (Z.-D.Z.); sunan1228@163.com (N.S.); xingquanzhu1@hotmail.com (X.-Q.Z.); 2 Taiyuan Zoo, Taiyuan 030009, China; liangliang20132017@163.com

**Keywords:** *Tetratrichomonas buttreyi*, *Pentatrichomonas hominis*, donkey, prevalence, zoonotic parasites, Shanxi Province

## Abstract

**Simple Summary:**

Trichomonads are among the most prevalent intestinal parasites with a worldwide distribution which can infect many animals, resulting in economic losses and threatening public health. The donkey raising industry in Shanxi Province is relatively well-developed; however, it is not yet known whether donkeys in Shanxi Province were infected with *Tetratrichomonas buttreyi* and *Pentatrichomonas hominis*. Thus, 815 fecal samples were collected from donkeys in three representative geographical locations in Shanxi Province to determine the prevalence and associated risk factors of *T. buttreyi* and *P. hominis* in donkeys using molecular approaches. The overall prevalence of *T. buttreyi* and *P. hominis* in donkeys in Shanxi Province was 25.4% and 0.7%, respectively. Genetic analysis revealed that all *P. hominis* sequences obtained in this study were identified as genotype CC1, suggesting possible zoonotic potential. This is the first report of *T. buttreyi* and *P. hominis* prevalence in donkeys worldwide, which not only extends the geographical distribution of trichomonads but also expands the host spectrum. The findings also have implications for the prevention and control of trichomonad infections in donkeys in Shanxi Province.

**Abstract:**

Two species of trichomonads, *Tetratrichomonas buttreyi* and *Pentatrichomonas hominis*, are common intestinal parasites that can impact animal health and productivity. Severe infection by these parasites can lead to diarrhea and wasting in affected animals. Notably, *P. hominis* is known to cause diarrhea and has the potential to be transmitted between animals and humans. Donkeys hold significant economic importance in China’s agricultural sector. However, whether donkeys are infected with *T. buttreyi* and *P. hominis* remains unknown globally. To address this gap in knowledge, 815 fecal samples were collected from donkeys in three representative regions in Shanxi Province, North China. Then, the presence and genetic characteristics of *T. buttreyi* and *P. hominis* were examined using species-specific PCR primers amplifying the small subunit ribosomal RNA genes. The overall prevalence was detected to be 25.4% (207/815) for *T. buttreyi* and 0.7% (6/815) for *P. hominis* in donkeys in Shanxi Province. All obtained *P. hominis* sequences were identified as genotype CC1. Genetic analysis revealed that all *P. hominis* isolates from donkeys were clustered into the same branch with isolates detected in humans, suggesting possible zoonotic transmission. This study is the first to report the occurrence and prevalence of *T. buttreyi* and *P. hominis* in donkeys globally. These findings expand the host range of trichomonads and improve our understanding of their genetic diversity and zoonotic potential, providing essential baseline data for the prevention and control of these parasites in donkeys in the region.

## 1. Introduction

*Tetratrichomonas buttreyi* and *Pentatrichomonas hominis* are two protozoan parasites of the Trichomonadidae family that inhabit the gastrointestinal tracts of humans and animals as parasites or commensals, posing significant public health challenges [1]. They exist in a trophozoite form, which is responsible for infection and replication within the intestines [2]. Both parasites have direct life cycles, with transmission occurring primarily through fecal–oral routes, and exhibit distinct characteristics and implications for human health [3,4].

In 1960, *T. buttreyi* was first identified in the ceca of pigs by Hibler et al. [5] and was considered a non-pathogenic commensal organism detected in pigs and cattle [2,6]. Recently, a report indicated that excessive infection by trichomonads can be pathogenic [7], and subsequently, symptoms such as diarrhea were observed in dairy cattle which were infected with *T. buttreyi* [4].

Existing evidence indicates that *P. hominis* is an opportunistic parasite causing diarrhea in humans, monkeys, dogs, pigs and cattle [8,9,10,11,12]. In addition, previous studies have shown that *P. hominis* may be recognized as a causative agent of diarrhea with potential for zoonotic transmission [13,14]. To date, most reports of *P. hominis* involve canids, with the prevalent genotypes detected in dogs being CC1, CC2 and CC3 [15]. It has also been sporadically reported in humans [16,17]. However, a previous study demonstrated that *P. hominis* infections may accelerate the development of colon cancer through changing gut microbiota [14]. With the deeper understanding of *P. hominis*, an increasing number of reports have indicated that *P. hominis* not only reproduces at the cecum or colon, but has also been detected in other organs, such as the anocelia [18,19].

Typically, microscopic examination is the routine method to discriminate trichomonad species. However, it is difficult to distinguish trichomonads due to their similar morphology under the microscope (e.g., *Trichomonas foetus* and *T. buttreyi*). With the rapid development of molecular detection methods, polymerase chain reaction (PCR)-based approaches have become important tools for detecting and identifying the trichomonads with higher specificity and sensitivity, especially in asymptomatic individuals [20,21,22]. The small subunit ribosomal RNA (SSU rRNA) gene is the main genetic marker to identify the species and genotypes of trichomonads [12].

The accurate identification of various trichomonad species is important for the diagnosis, treatment and surveillance of trichomonad infections in humans and animals. Ronidazole is a potentially neurotoxic drug, used for the treatment of feline trichomoniasis caused by *Trichomonas foetus* infection [23]. Metronidazole is considered the drug of choice for the treatment of *P. hominis*; however, it is proven to be ineffective against *Trichomonas foetus* [3]. Therefore, the accurate identification of trichomonad species is necessary to establish the correct treatment plan.

China is among the top countries in donkey breeding in the world. Historically, donkeys have been valuable for trade and are now appreciated for their nutritional benefits [24]. Donkeys play a significant economic role in rural areas, providing tender meat, nutritious skin, and milk [25,26,27]. Due to the growing significance of trichomonads in veterinary medicine, an increasing number of studies have been conducted on the prevalence and pathogenicity of trichomonad infections in different vertebrates. However, no studies have been published on the epidemiology of *T. buttreyi* and *P. hominis* in donkeys globally. Thus, this study firstly investigated the occurrence, prevalence and genetic characterization of *T. buttreyi* and *P. hominis* in donkeys in Shanxi Province, expanding the host spectrum and providing the baseline data to control and prevent these parasites in the study areas.

## 2. Materials and Methods

### 2.1. Sampling Collection

From April to May 2023, 815 fresh fecal specimens were sampled from donkeys in three representative cities in Shanxi Province: 81 from Jinzhong city, 363 from Linfen city and 371 from Datong city. To minimize contamination, the uppermost part of each freshly excreted fecal sample was collected using a disposable glove and recorded with relevant details, including region, sex and age. The donkey feces were categorized into two age groups (donkeys aged three years and above, and those which were lower than 3 years) and two sex groups (male and female). All samples were then transported under cool conditions to the Laboratory of Parasitic Diseases, College of Veterinary Medicine, Shanxi Agricultural University, and they were stored at −20 °C until needed for PCR-based molecular analysis.

### 2.2. DNA Extraction and PCR Amplification

Following the instructions provided in the E.Z.N.A.^®^ Stool DNA Kit (Omega Biotek, Inc., Norcross, GA, USA), genomic DNA was extracted from approximately 200 mg of each fecal sample and then stored at −20 °C until PCR amplification. A total of 25 μL PCR mixture was prepared, including 2 µL of dNTPs, 2.5 µL of 10× PCR Buffer (Mg^2+^ free), 25 mM of MgCl_2_, 1.25 U of *Ex*-Taq (Takara, Dalian, China), 1 µL of each primer, 2 µL of genomic DNA and 14.75 µL of ddH_2_O. The PCR primers and amplification procedures referred to previous studies [8,28] and are listed in Table 1. Each PCR assay included both negative controls (reagent-grade water) and positive controls (verified DNA of *T. buttreyi* or *P. hominis* by sequencing) to ensure the reliability of the results. The amplicons were analyzed on 1.5% agarose gels containing ethidium bromide and observed using UV transillumination, and the positive ones were sequenced by Sangon Biotech Co., Ltd. (Shanghai, China) bidirectionally.

### 2.3. Sequencing and Phylogenetic Analysis

In this study, we utilized Chromas V2.6 software to proofread and assemble the obtained sequences; then, the Basic Local Alignment Search Tool (BLAST) was subsequently used to identify species by alignment with relevant sequences of known species available in the GenBank database. A phylogenetic analysis was conducted with the Neighbor-joining (NJ) method in MEGA 7.0 software, applying the Kimura-2-parameter model. To evaluate the robustness of the reconstructed phylogenetic trees, we performed a bootstrap analysis with 1000 replicates.

### 2.4. Statistical Analysis

The chi-square (χ2) test was used to evaluate the relevance between the prevalence of *T. buttreyi* or *P. hominis* across various regions, ages and sexes, employing SPSS 26.0 software (SPSS Inc., Chicago, IL, USA). Moreover, odds ratios (ORs) and 95% confidence intervals (95% CIs) were calculated to determine the strength of the correlation between prevalence and the examined variables. A *p*-value of less than 0.05 was considered to be statistically significant.

## 3. Results

### 3.1. Prevalence of T. buttreyi and P. hominis in Donkeys

In this study, 207 out of 815 fecal samples and 6 out of 815 fecal samples from donkeys were detected as *T. buttreyi*- and *P. hominis*-positive, respectively. The overall prevalence in Shanxi Province was 25.4% for *T. buttreyi* (95% CI: 22.4–28.4) and 0.7% for *P. hominis* (95% CI: 0.2–1.3), respectively (Table 2). Among the donkeys in the three cities examined, donkeys in Linfen city had the highest *T. buttreyi* prevalence of 31.7% (115/363), while donkeys in Datong city had the highest *P. hominis* prevalence of 1.1% (4/371). Statistical analysis showed that significant differences in the prevalence of *T. buttreyi* were observed in donkeys among region groups (*p* < 0.001) and sex groups (*p* < 0.001). In contrast to *T. buttreyi*, no statistically significant difference was found in donkeys between region groups and sex groups in the prevalence of *P. hominis* (*p* > 0.05). However, a statistically significant difference in *P. hominis* prevalence (*p* < 0.05) was found between donkeys aged ≥3 years (0.3%, 2/601) and donkeys aged <3 years (1.9%, 4/214). Additionally, among the 815 fecal samples, the co-infection of both *T. buttreyi* and *P. hominis* was detected in a female donkey in Datong city which was aged less than 3 years, with no clinical symptoms.

### 3.2. Sequence Analysis of T. buttreyi and P. hominis 

*T. buttreyi*-positive samples were sequenced, and nine distinct sequence types showing 98.1–99.8% sequence similarity were obtained. Among the 207 *T. buttreyi* sequences obtained from donkeys in this study, 129, 64 and 8 sequences were identical to the reported *T. buttreyi* sequences in China with accession numbers PP256577 (pig), PP256576 (pig) and MK880285 (cattle), respectively. Six other sequences showed 98.4–99.8% identity to the reported *T. buttreyi* sequence (accession number: PP256576) isolated from pigs in Shanxi Province.

Regarding the obtained 6 sequences of *P. hominis* in this study, comparative analysis showed that 66.7% (4/6) of these sequences had 100% similarity to the reported *P. hominis* sequence isolated from a fox in China (accession number: OM763804), and another 2 sequences exhibited 99.7% homology with reference sequences isolated from dogs in China (KX136890 and KX136876), respectively. In addition, all *P. hominis* sequences obtained from donkeys in this study were identified as genotype CC1.

### 3.3. Phylogenetic Analysis of T. buttreyi and P. hominis

**Figure 1:**
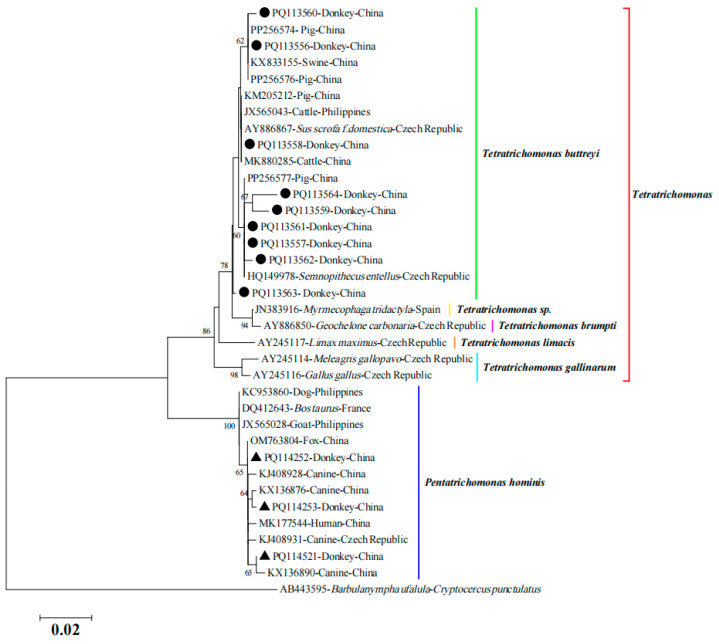
Phylogenetic relationship of trichomonad species inferred from SSU rRNA gene sequences using Neighbor-joining analysis, based on Kimura two-parameter model, with 1000 bootstrap replications. *T. buttreyi* sequences obtained in this study are marked with black circle (●) and those of *P. hominis* (▲) are marked with black triangle. Bootstrap values are shown when >50%.

To better understand the genetic relationship of *T. buttreyi* and *P. hominis* detected in this study, a phylogenetic tree was reconstructed including other related trichomonad species. As shown in Figure 1, sequences of *T. buttreyi* and *P. hominis* from this study were clustered with reported animal-derived sequences. Notably, the three representative sequences of *P. hominis* from donkeys also clustered with a *P. hominis* sequence isolated from a human, indicating potential zoonotic transmission. The representative sequences from this study were deposited in the GenBank database with the following accession numbers: PQ113556 to PQ113564 for *T. buttreyi* and PQ114251 to PQ114253 for *P. hominis*.

## 4. Discussion

*T. buttreyi* and *P. hominis* are parasitic protozoans that commonly inhabit the intestinal tracts of various vertebrates. Notably, *P. hominis* has been verified as a zoonotic parasite infecting a number of mammals such as humans, primates, cats, dogs and cattle, causing serious gastrointestinal symptoms [18,29,30]. The trophozoite stage of *P. hominis* can form a pseudocyst under adverse conditions and can survive outside the host for several days, thereby increasing the risk of infection to other hosts [10,30]. Up to now, no studies have reported the occurrence of *T. buttreyi* and *P. hominis* in donkeys globally. Thus, the present study first examined the occurrence and genetic characterization of *T. buttreyi* and *P. hominis* in donkeys.

In the present study, the prevalence of *T. buttreyi* in donkeys in Shanxi Province was 25.4% (207/815), which was higher than the average prevalence in cattle in China [31] and lower than that in pigs in other provinces of China [6] and some other countries, e.g., the Philippines [32]. Interestingly, a recent study reported a significantly higher prevalence of *T. buttreyi* in pigs (49.7%, 180/362) in Shanxi Province [33]. These differences in *T. buttreyi* prevalence might be influenced by factors such as geographic location, animal species, age distribution, feeding and management practices, ecological conditions, sex composition and the immune status of the animals. Further studies sampling larger numbers of animals and diverse animal species are needed to better understand the factors influencing the prevalence of *T. buttreyi* in different animals. 

Shanxi Province, characterized by a loess-covered mountainous plateau, experiences significant variations in precipitation due to its topography, with annual rainfall ranging from 358 to 621 mm [34]. The highest prevalence of *T. buttreyi* in donkeys in this study was observed in Linfen city, which is located in the southern part of Shanxi Province and has higher humidity compared to other cities. A previous report indicated that trichomonads can survive for several days in moist environments [1]. Thus, we speculate that the favorable temperature and humidity in Linfen city contribute to the higher prevalence of *T. buttreyi* in donkeys. Additionally, the prevalence of *T. buttreyi* in donkeys in this study showed an age-dependent increase, which is not consistent with a previous report in pigs in China [8]. Statistical analysis also showed significant differences in *T. buttreyi* prevalence among sex groups (*p* < 0.001), with female donkeys showing a higher prevalence. Also, sex has been identified as a risk factor for trichomonad infection in non-human primates in China [35]. 

Based on SSU rRNA gene sequences of *P. hominis*, the prevalence of *P. hominis* in donkeys in Shanxi Province was 0.7% (6/815, 95% CI: 0.2–1.3). Notably, *P. hominis* was found in all regions except Jinzhong city. With regard to the age groups, a statistically significant difference in the prevalence of *P. hominis* was observed in the examined donkeys, and donkeys aged <3 years had a 5.7 times higher risk of infection compared with those aged ≥3 years. Previous studies also suggest that age is a critical factor in *P. hominis* transmission among animals and humans, but more epidemiological investigations are required to reveal the risk factors affecting the prevalence of *P. hominis* infection in different hosts, and to elucidate the pathogenic potential of *P. hominis* in young donkeys [31,36]. Generally, younger animals are more susceptible to parasites due to their less developed immune systems.

The gut microbiota, a complex ecosystem within the host, is essential for maintaining immune and metabolic homeostasis [37,38]. Studies have shown that infections with many gastrointestinal parasites often disrupt this balance, impacting host health [36]. *P. hominis* infection in female foxes, for instance, has been linked to gut microbiota imbalances, diarrhea and wasting symptoms [15]. Moreover, *P. hominis* can exacerbate colon cancer by altering patients’ gut microbiota [14,39].

Close connections between hosts and through fecal–oral routes via the ingestion of trophozoites are considered routes of *P. hominis* transmission [36]. In recent years, *P. hominis* has been identified in the feces of felines and canids, and in economic animals such as cattle [30], pigs [8] and goats [40], suggesting that these animals can act as reservoirs for further transmission [41]. Overall, six sequences obtained in this study were identified as genotype CC1, which was frequently detected in canids, e.g., dogs, foxes and raccoon dogs [15,30]. Notably, the genotype CC1 was also reported in Siberian tigers (*Panthera tigris altaica*) [28], dogs [41], monkeys [36], goats [40], foxes [15] and humans [36] in China, indicating that this genotype is not host-specific and suggesting potential zoonotic transmission of *P. hominis* between different hosts. Dogs present on donkey farms may contribute to the *P. hominis* infection of donkeys, though the transmission between donkeys and dogs remains unclear. In addition, *P. hominis* has been detected in wild animals like the boa (*Boa constrictor imperator)* and the Philippine scops owl (*Otus megalotis)*, suggesting its wide host spectrum and potential health risks to both humans and animals [32].

Phylogenetic analysis indicated that the six sequences of *P. hominis* obtained from donkeys in this study were clustered into one branch containing known *P. hominis* sequences identified in humans, suggesting potential zoonotic transmission. The present study used SSU rRNA sequences as genetic markers for the identification of *T. buttreyi* and *P. hominis*. However, SSU rRNA sequences have limitations as genetic markers for the differentiation of closely related species and/or cryptic species [42,43]. Thus, more appropriate genetic markers, such as the internal transcribed spacers (ITS-1 and ITS-2) and mitochondrial cytochrome oxidase subunit I (*cox*1), should be used for the precise identification and accurate differentiation of closely related species and/or cryptic species [42,43]. Notably, no diarrhea symptoms were observed in the positive donkeys, and all had normal stool consistency. Therefore, further research is needed to confirm the pathogenicity of *P. hominis* infection in donkeys. This study not only addresses the knowledge gap of *T. buttreyi* and *P. hominis* infection in donkeys worldwide, but also provides useful information for implementing measures to control *T. buttreyi* and *P. hominis* infections in donkeys in the studied areas.

## 5. Conclusions

This study revealed that the prevalence of *T. buttreyi* and *P. hominis* in donkeys in Shanxi Province was 25.4% and 0.7%, respectively. Genetic analysis identified the CC1 genotype of *P. hominis* in these donkeys, suggesting that donkeys might serve as a potential host for *P. hominis* transmission. To our knowledge, this is the first report of the occurrence and prevalence of *T. buttreyi* and *P. hominis* in donkeys globally, which not only extends the host range of *T. buttreyi* and *P. hominis*, but also highlights the public health significance of *P. hominis*. 

## Figures and Tables

**Table 1:** PCR primers and parameters used in this study.

Species	Gene	Primer ID	Primer Sequences (5′-3′)	Annealing Temperatures (°C)	Fragment Length (bp)
*T. buttreyi*	SSU rRNA	FF	GCGCCTGAGAGATAGCGACTA	59	
		RR	GGACCTGTTATTGCTACCCTCTTC		
		bF	GTTTTTTCTCAGGCAGCAATG	61	623
		bR	GCAACCTAGAAACCTAGGCG		
*P. hominis*	SSU rRNA	F1	ATGGCGAGTGGTGGAATA	60	
		R1	CCCAACTACGCTAAGGATT		
		F2	TGTAAACGATGCCGACAGAG	60	339
		R2	CAACACTGAAGCCAATGCGAGC		

**Table 2:** Factors associated with prevalence of *T. buttreyi* and *P. hominis* in donkeys in Shanxi Province, North China.

Species	Factor	Category	No. Positive/No. Tested	Prevalence % (95% CI)	OR (95% CI)	*p*-Value
*T. buttreyi*	Region	Jinzhong	16/81	19.8 (11.1–28.4)	Ref.	<0.001
		Linfen	115/363	31.7 (26.9–36.5)	1.9 (1.0–3.4)	
		Datong	76/371	20.5 (16.4–24.6)	1.1 (0.6–1.9)	
	Age	≥3 years	160/601	26.6 (23.1–30.2)	1.3 (0.9–1.9)	0.179
		<3 years	47/214	22.0 (16.4–27.5)	Ref.	
	Sex	Male	15/120	12.5 (6.6–18.4)	Ref.	<0.001
		Female	192/695	27.6 (24.3–31.0)	2.7 (1.5–4.7)	
		Sub-total	207/815	25.4 (22.4–28.4)		
						
*P. hominis*	Region	Jinzhong	0/81	0		0.428
		Linfen	2/363	0.6 (0.0–1.3)	Ref.	
		Datong	4/371	1.1 (0.0–2.1)	2.0 (0.4–10.8)	
	Age	≥3 years	2/601	0.3 (0.0–0.8)	Ref.	0.024
		<3 years	4/214	1.9 (0.1–3.7)	5.7 (1.0–31.4)	
	Sex	Male	2/120	1.7 (0.0–4.0)	2.9 (0.5–16.2)	0.197
		Female	4/695	0.6 (0.0–1.1)	Ref.	
		Sub-total	6/815	0.7 (0.2–1.3)		

## Data Availability

The data sets supporting the results of this article have been submitted to GenBank, and the accession number is shown in the article.

## References

[1] Gookin J.L., Hanrahan K., Levy M.G. (2017). The conundrum of feline trichomonosis. J. Feline Med. Surg..

[2] Mostegl M.M., Richter B., Nedorost N., Maderner A., Dinhopl N., Kulda J., Liebhart D., Hess M., Weissenböck H. (2010). Design and validation of an oligonucleotide probe for the detection of protozoa from the order trichomonadida using chromogenic in situ hybridization. Vet. Parasitol..

[3] Bastos B.F., Brener B., de Figueiredo M.A., Leles D., Mendes-de-Almeida F. (2018). *Pentatrichomonas hominis* infection in two domestic cats with chronic diarrhea. JFMS Open. Rep..

[4] Castella J., Muńoz E., Ferrer D., Gutiérrez J.F. (1997). Isolation of the trichomonad *Tetratrichomonas buttreyi* (Hibler et al., 1960) Honigberg, 1963 in bovine diarrhoeic faeces. Vet. Parasitol..

[5] Rivera W.L., Lupisan A.J., Baking J.M. (2008). Ultrastructural study of a tetratrichomonad isolated from pig fecal samples. Parasitol. Res..

[6] Li W.C., Wang K., Li Y., Zhao L.P., Xiao Y., Gu Y.F. (2018). Survey and molecular characterization of trichomonads in pigs in Anhui Province, East China, 2014. Iran. J. Parasitol..

[7] Mostegl M.M., Richter B., Nedorost N., Maderner A., Dinhopl N., Weissenböck H. (2011). Investigations on the prevalence and potential pathogenicity of intestinal trichomonads in pigs using in situ hybridization. Vet. Parasitol..

[8] Li W., Li W., Gong P., Zhang C., Yang J., Zhang X., Li J. (2015). The prevalence of intestinal trichomonads in Chinese pigs. Vet. Parasitol..

[9] Cobo E.R., Corbeil L.B., Agnew D.W., VanHoosear K., Friend A., Olesen D.R., BonDurant R.H. (2007). *Tetratrichomonas* spp. and *Pentatrichomonas hominis* are not persistently detectable after intravaginal inoculation of estrous heifers. Vet. Parasitol..

[10] Meloni D., Mantini C., Goustille J., Desoubeaux G., Maakaroun-Vermesse Z., Chandenier J., Gantois N., Duboucher C., Fiori P.L., Dei-Cas E. (2011). Molecular identification of *Pentatrichomonas hominis* in two patients with gastrointestinal symptoms. J. Clin. Pathol..

[11] Fukushima T., Mochizuki K., Yamazaki H., Watanabe Y., Yamada S., Aoyama T., Sakurai Y., Mori H., Nakazawa M. (1990). *Pentatrichomonas hominis* from beagle dogs—Detection method, characteristics and route of infection. Exp. Anim..

[12] Gookin J.L., Stauffer S.H., Levy M.G. (2007). Identification of *Pentatrichomonas hominis* in feline fecal samples by polymerase chain reaction assay. Vet. Parasitol..

[13] Abdo S.M., Ghallab M.M.I., Elhawary N.M., Elhadad H. (2022). *Pentatrichomonas hominis* and other intestinal parasites in school-aged children: Coproscopic survey. J. Parasit. Dis..

[14] Zhang H., Yu Y., Li J., Gong P., Wang X., Li X., Cheng Y., Yu X., Zhang N., Zhang X. (2022). Changes of gut microbiota in colorectal cancer patients with *Pentatrichomonas hominis* infection. Front. Cell. Infect. Microbiol..

[15] Song P., Guo Y., Zuo S., Li L., Liu F., Zhang T., Dai H., Dong H. (2023). Prevalence of *Pentatrichomonas hominis* in foxes and raccoon dogs and changes in the gut microbiota of infected female foxes in the Hebei and Henan Provinces in China. Parasitol. Res..

[16] Zhang N., Zhang H., Yu Y., Gong P., Li J., Li Z., Li T., Cong Z., Tian C., Liu X. (2019). High prevalence of *Pentatrichomonas hominis* infection in gastrointestinal cancer patients. Parasit. Vectors.

[17] Phuanukoonnon S., Michael A., Kirarock W.S., Pomat W.S., van den Biggelaar A.H. (2013). Intestinal parasitic infections and anaemia among pregnant women in the highlands of Papua New Guinea. Papua New Guin. Med. J..

[18] Maritz J.M., Land K.M., Carlton J.M., Hirt R.P. (2014). What is the importance of zoonotic trichomonads for human health?. Trends Parasitol..

[19] Dong N., Jin X., Huang J., Chen K., Li Y., Chen C., Hu D., Xie Y. (2019). Tetratrichomonas in pyopneumothorax. Am. J. Emerg. Med..

[20] Cobo E.R., Campero C.M., Mariante R.M., Benchimol M. (2003). Ultrastructural study of a tetratrichomonad species isolated from prepucial smegma of virgin bulls. Vet. Parasitol..

[21] Baltrušis P., Höglund J. (2023). Digital PCR: Modern solution to parasite diagnostics and population trait genetics. Parasit. Vectors.

[22] Maurer J.J. (2011). Rapid detection and limitations of molecular techniques. Annu. Rev. Food. Sci. Technol..

[23] Gookin J.L., Copple C.N., Papich M.G., Poore M.F., Stauffer S.H., Birkenheuer A.J., Twedt D.C., Levy M.G. (2006). Efficacy of ronidazole for treatment of feline *Tritrichomonas foetus* infection. J. Vet. Intern. Med..

[24] Davis E. (2019). Donkey and mule welfare. Vet. Clin. N. Am. Equine. Pract..

[25] Seyiti S., Kelimu A. (2021). Donkey industry in China: Current aspects, suggestions and future challenges. J. Equine Vet. Sci..

[26] Wang X., Wang T., Liang H., Wang L., Akhtar F., Shi X., Ren W., Huang B., Kou X., Chen Y. (2023). A novel SNP in NKX1-2 gene is associated with carcass traits in Dezhou donkey. BMC Genom. Data.

[27] Yu M., Zhang X., Yan J., Guo J., Zhang F., Zhu K., Liu S., Sun Y., Shen W., Wang J. (2022). Transcriptional specificity analysis of testis and epididymis tissues in Donkey. Genes.

[28] Zhang H., Zhang N., Gong P., Cheng S., Wang X., Li X., Hou Z., Liu C., Bi T., Wang B. (2022). Prevalence and molecular characterization of *Pentatrichomonas hominis* in Siberian tigers (*Panthera tigris altaica*) in northeast China. Integr. Zool..

[29] Bailey N.P., Velo-Rego E., Hirt R.P. (2021). Sporadic isolation of tetratrichomonas species from the cattle urogenital tract. Parasitology.

[30] Mahittikorn A., Udonsom R., Koompapong K., Chiabchalard R., Sutthikornchai C., Sreepian P.M., Mori H., Popruk S. (2021). Molecular identification of *Pentatrichomonas hominis* in animals in central and western Thailand. BMC Vet. Res..

[31] Li W.C., Huang J.M., Fang Z., Ren Q., Tang L., Kan Z.Z., Liu X.C., Gu Y.F. (2020). Prevalence of *Tetratrichomonas buttreyi* and *Pentatrichomonas hominis* in yellow cattle, dairy cattle, and water buffalo in China. Parasitol. Res..

[32] Dimasuay K.G., Rivera W.L. (2013). Molecular characterization of trichomonads isolated from animal hosts in the Philippines. Vet. Parasitol..

[33] Wang Z.R., Fan Q.X., Wang J.L., Zhang S., Wang Y.X., Zhang Z.D., Gao W.W., Zhu X.Q., Liu Q. (2024). Molecular identi-fication and survey of Trichomonad species in pigs in Shanxi Province, North China. Vet. Sci..

[34] Gao W.W., Zhang S., Zhang T.H., Xiao H.D., Su N., Tao M.F., Wu Z.X., Zhang Z.D., Zhu X.Q., Xie S.C. (2023). Prevalence and multilocus genotyping of *Giardia duodenalis* in donkeys in Shanxi Province, North China. Animals.

[35] Ma P.P., Zou Y., Mu W.J., Zhang Y.Y., Li Y.Q., Liu Z.L., Zhang L., Chen L.X., Liu G.H., Wang S. (2024). Prevalence of in-testinal trichomonads in captive non-human primates in China. Parasite.

[36] Li W.C., Ying M., Gong P.T., Li J.H., Yang J., Li H., Zhang X.C. (2016). *Pentatrichomonas hominis*: Prevalence and molecular characterization in humans, dogs, and monkeys in Northern China. Parasitol. Res..

[37] Peachey L.E., Castro C., Molena R.A., Jenkins T.P., Griffin J.L., Cantacessi C. (2019). Dysbiosis associated with acute helminth infections in herbivorous youngstock - observations and implications. Sci. Rep..

[38] Thursby E., Juge N. (2017). Introduction to the human gut microbiota. Biochem. J..

[39] Dheilly N.M., Ewald P.W., Brindley P.J., Fichorova R.N., Thomas F. (2019). Parasite-microbe-host interactions and cancer risk. PLoS Pathog..

[40] Li W.C., Wang K., Gu Y. (2018). Occurrence of *Blastocystis* sp. and *Pentatrichomonas hominis* in sheep and goats in China. Parasit. Vectors.

[41] Li W.C., Gong P.T., Ying M., Li J.H., Yang J., Li H., Yang Z.T., Zhang G.C., Zhang X.C. (2014). *Pentatrichomonas hominis*: First isolation from the feces of a dog with diarrhea in China. Parasitol. Res..

[42] Ghafar A., Abbas G., Beasley A., Bauquier J., Wilkes E.J.A., Jacobson C., McConnell E., El-Hage C., Carrigan P., Cudmore L. (2023). Molecular diagnostics for gastrointestinal helminths in equids: Past, present and future. Vet. Parasitol..

[43] Akhoundi M., Downing T., Votýpka J., Kuhls K., Lukeš J., Cannet A., Ravel C., Marty P., Delaunay P., Kasbari M. (2017). Leishmania infections: Molecular targets and diagnosis. Mol. Aspects Med..

